# Reproducibility of ^18^F-Sodium Fluoride Positron Emission Tomography for Assessing Microcalcification in Coronary Arterial and Thoracic Aortic Atherosclerosis: Is the Signal below the Resolution of PET?

**DOI:** 10.1007/s11886-025-02240-9

**Published:** 2025-05-14

**Authors:** Ondrej Fanta, Shiv Patil, Thomas Werner, Drew A. Torigian, Abass Alavi

**Affiliations:** Department of Radiology, University of Pennsylvania, 3400 Spruce Street, Philadelphia, PA 19107 USA

**Keywords:** Atherosclerosis, Microcalcification, Coronary artery disease, ^18^F-Sodium fluoride, Positron emission tomography (PET), Spatial resolution

## Abstract

**Purpose of Review:**

The rising prevalence of atherosclerosis has prompted the development of novel diagnostic methods capable of identifying early-stage disease when therapeutic interventions may be most effective. ^18^F-sodium fluoride (NaF) positron emission tomography/computed tomography (PET/CT) is a molecular imaging technique that can quantify subclinical microcalcification in arterial plaque. The focus of this review article is to discuss the utility of ^18^F-NaF PET/CT in assessing atherosclerotic disease of major susceptible blood vessels, particularly the coronary arteries and thoracic aorta.

**Recent Findings:**

^18^F-NaF uptake observed on PET imaging demonstrates promising potential as a marker of atherosclerotic burden in individual coronary arteries, whole heart segmentations, and the thoracic aorta. Global versus focal assessment of ^18^F-NaF uptake in small arteries is a significant source of methodological heterogeneity among studies.

**Summary:**

The accuracy and reproducibility of ^18^F-NaF PET/CT may be improved by standardized quantification methods in light of the limited spatial resolution of PET, particularly through the use of techniques to evaluate global atherosclerotic burden.

## Introduction

Atherosclerosis is a chronic cardiovascular disease (CVD) that is a leading cause of morbidity and mortality worldwide [1]. In 2022, 19.8 million deaths were attributable to complications of CVD such as ischemic heart disease and stroke, equating to 396 million years of lives lost and 44.9 million years lived with disability [2]. The pathogenesis of atherosclerosis is characterized by the gradual accumulation of lipid-laden plaques along the walls of blood vessels, thereby compromising perfusion and posing a thrombotic risk if ruptured [3]. An initial stressor, such as hypertension and intimal lipid retention, leads to vascular endothelial dysfunction that induces localized inflammation and initial formation of the atheroma [4]. Chronic plaque inflammation promotes mineralization via microcalcification and eventual aggregation into macroscopic calcification [5].

The cascade of plaque development occurs perniciously as an asymptomatic process that contributes to adverse cardiovascular events over time. This has necessitated efforts to improve methods of identifying vulnerable patient populations and thus enable clinical interventions for early-stage disease [6]. Imaging modalities such as conventional angiography and computed tomography (CT) are routinely used to determine the severity of atherosclerotic disease [7]. However, these approaches predominantly reveal plaque macrocalcifications that manifest in late-stage disease and provide limited information on early-stage atheroma formation.

**Fig. 1:**
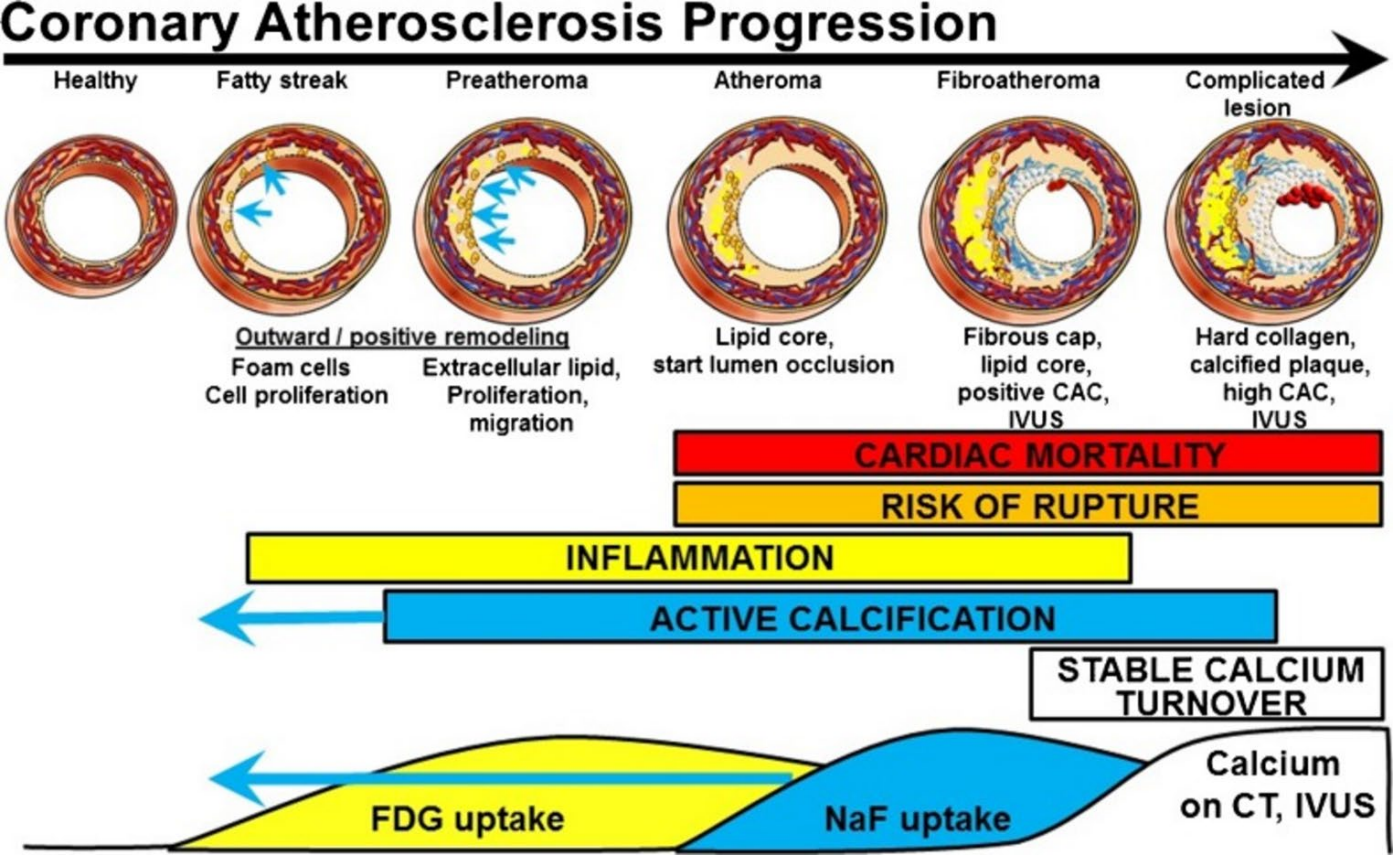
Atherosclerosis progression and possible visualization with different imaging modalities. The figure depicts the major advantage of 18 F-NaF over more conventional imaging used in atherosclerosis. (Reproduced from: McKenney-Drake ML, et al. Eur J Nucl Med Mol Imaging. 2018 Nov;45 [12]:2190–200, with permission from Springer Nature) [10]

Positron emission tomography (PET) is a molecular imaging technique capable of visualizing biologically active processes that are undetectable on standard structural imaging techniques [8]. In particular, the inflammation and microcalcification characteristic of subclinical plaque formation can be detected by ^18^F-fluorodeoxyglucose (FDG) and ^18^F-sodium fluoride (NaF), respectively [9] (Fig. 1). ^18^F-NaF offers several advantages over ^18^F-FDG, including less spillover from adjacent tissue structures, which is particularly relevant for myocardial uptake of ^18^F-FDG, and faster blood clearance than ^18^F-FDG. The reduced blood pool activity observed with ^18^F-NaF PET imaging makes quantification of arterial wall radiotracer uptake significantly more accurate [10].

Yet, the fundamental weakness that needs to be accounted for is the relatively low spatial resolution of PET, which causes a partial volume effect [11]. This is the result of the inherent physical properties of positron emitting radiotracers, the limits of accuracy of current detector technology, and the techniques used to produce PET images. This is particularly problematic when measuring small structures of interest, a prime example of which is the coronary artery, which in addition to being only around 2–4 mm in diameter for the major vessels, also moves during respiration and cardiac contractions, further blurring the radiotracer uptake [12].

The aim of this article is to provide a critical review of the clinical application of PET for assessing microcalcification in coronary arterial and thoracic aortic atherosclerosis. An overview of the major original research studies in this domain as well as consideration of the strengths and limitations of PET, including issues related to spatial resolution, are discussed.

## Imaging Atherosclerosis with ^18^F-NaF PET

The visualization of atherosclerosis by ^18^F-NaF PET has been investigated since the early 2010s, when studies demonstrated that vascular ^18^F-NaF uptake is strongly correlated with CVD risk factors [13–17]. Joshi et al. demonstrated the ^18^F-NaF avidity of high risk and ruptured atherosclerotic plaques retrieved during carotid endarterectomy [18]. Several studies compared ^18^F-NaF PET/CT to other imaging modalities, demonstrating sites of increased ^18^F-NaF uptake with few CT calcifications. In a study of 75 patients who underwent ^18^F-NaF PET/CT for the exclusion of osseous metastases, Derlin et al. found that 88% of lesions with radiotracer uptake had associated calcification on CT, but that only 12% of calcifications visible on CT had associated increased ^18^F-NaF uptake on PET, where the ^18^F-NaF accumulation was higher in more extensive calcifications than smaller deposits [19]. This finding, which has been reproduced in numerous studies, indicates the ability of ^18^F-NaF to discern plaques with active calcification from those with stable calcification.

Animal studies performed in an Ossabaw miniature swine metabolic syndrome model and lean controls by McKenney-Drake et al. compared ^18^F-NaF uptake on PET with CT, intravascular ultrasonography (IVUS), and histopathology results [20]. Atherosclerotic lesions, in the left anterior descending coronary artery and right coronary artery, with high ^18^F-NaF uptake in animals with metabolic syndrome showed a strong correlation with percent wall coverage observed on IVUS (a method to quantify intimal thickening), which in turn correlated with histopathology findings using Verhoeff–van Gieson staining (a method to reveal elastic fibers and that is useful for visualizing pathologies in elastic arteries [21], *r* = 0.93, *p* < 0.0001). The metabolic syndrome group also had an Alavi-Carlsen calcification score (ACS, a global measure of atherosclerotic burden via ^18^F-NaF PET equal to the average cardiac mean standardized uptake value (SUVmean)) of 2.5 times greater than that in lean controls (*p* < 0.05). Neither group had any visible CT calcifications, demonstrating the ability of ^18^F-NaF PET to visualize atherosclerotic calcification before it is detectable on CT.

Early research of ^18^F-NaF demonstrated the usefulness of this radiotracer to detect atherosclerotic calcification earlier than CT and the possibility of detecting high risk plaques and plaques with active calcification. This made it a promising area for prospective studies of coronary artery disease and other related atherosclerotic disorders.

### PET/CT Spatial Resolution

**Fig. 2:**
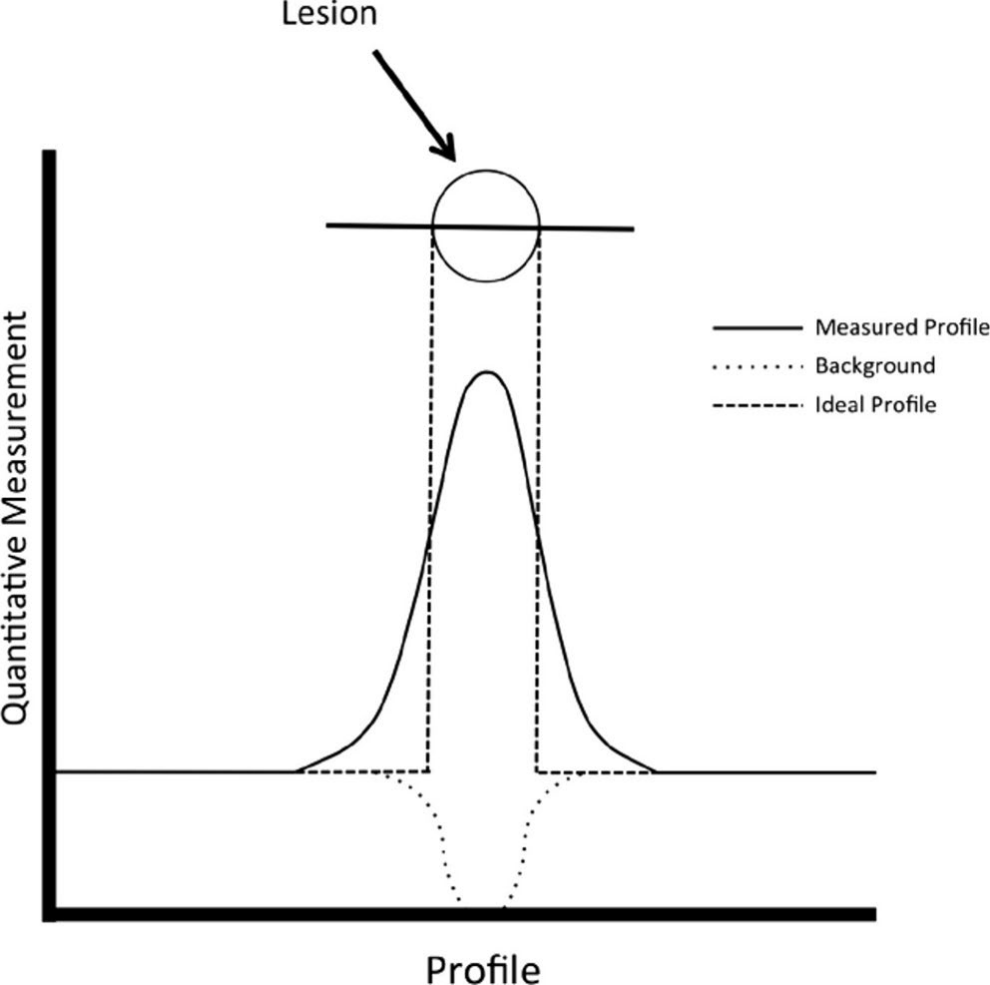
The figure depicts the ideal and measured profiles of a spherical lesion; unlike the ideal profile the measured profile has an unclear boundary, demonstrating the partial volume effect. If the difference between the activity of the lesion and background is not large enough, the lesion might become impossible to accurately measure. (Reproduced from: Houshmand S., et al. Pet Clinics. 2015 Jan; with permission from Elsevier) [28]

The spatial resolution of PET is a critical consideration for the analysis of small vessels. Several factors, including detector size, positron range, and image reconstruction quality, influence the range of PET’s spatial resolution [22]. The inherent physical properties of the PET radiotracer (i.e., positron endpoint energy), along with the density of human tissue, determines the positron range. In the case of ^18^F, the Gaussian blurring caused by the positron range is 0.54 mm full width half maximum (FWHM). There are constant efforts to improve spatial resolution, mostly using image processing, but also with the advancement of detectors. Current spatial resolution in the uEXPLORER PET/CT system (United Imaging, Houston, TX, USA) ranges from 3 to 4.7 mm FWHM (radial distance 1 to 20 cm) measured using phantoms [23]. This is a great improvement compared to earlier PET machines, including for example the spatial resolution of the Biograph mCT Flow 64-4R PET/CT system (Siemens Healthineers, Groningen, The Netherlands) which ranges from 4.3 to 7.8 mm FWHM (radial distance 1 to 20 cm) [24]. However, the resolution of current PET/CT machines still reaches a grey zone in small structures such as the coronary arteries, especially when measuring low differences between the target and surrounding tissue [25–28] (Fig. 2).

### ^18^F-NaF PET for Coronary Arterial Atherosclerosis

Atherosclerosis is a major cause of coronary artery disease (CAD). Conventional imaging methods include fluoroscopic coronary arterial angiography to visualize luminal stenosis/occlusion, non-contrast-enhanced coronary CT to assess coronary arterial calcium score, contrast-enhanced CT angiography to assess coronary arterial atherosclerotic plaque and luminal stenosis, or other structural imaging modalities such as coronary magnetic resonance angiography (MRA). However, these approaches have notable limitations in their ability to quantify coronary atheroburden. Calcium scores can show progression of atherosclerosis but only detect macrocalcifications that appear in the late stages of the disease process, thus limiting the utility for early intervention. Fluoroscopic coronary angiography remains the reference standard for detecting luminal stenosis/occlusion in need of intervention but is not useful for assessing the composition of plaque or for determining the true extent of coronary arterial atherosclerosis since arteries have a remarkable capacity for eccentric growth, meaning that even large plaques can have little or no effect on blood flow [29]. Microcalcification assessed by ^18^F-NaF PET/CT begins far earlier in the disease process and as such demonstrates promise in quantifying clinically actionable atherosclerosis prior to symptom onset [10].

**Fig. 3:**
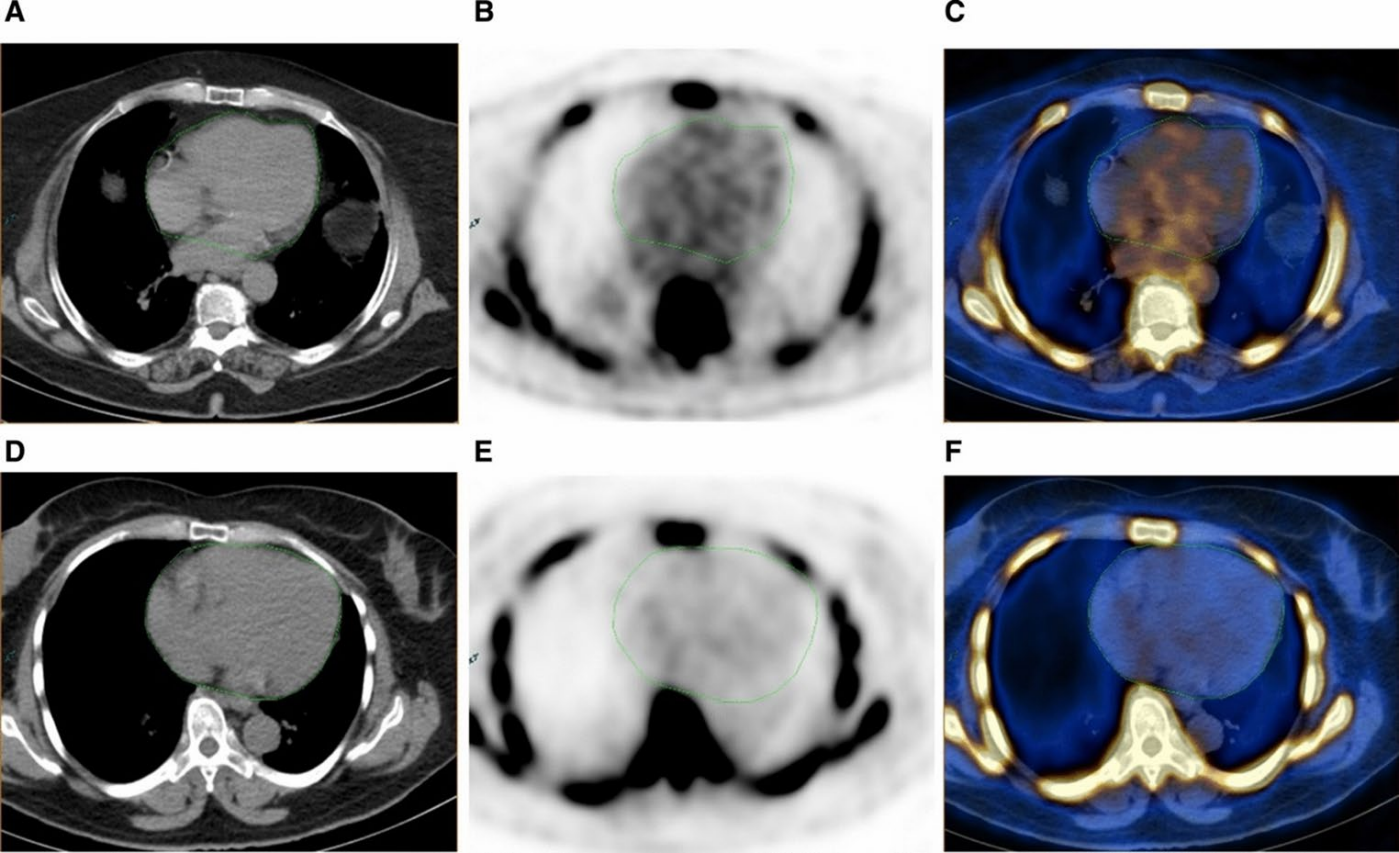
Subject with high global molecular calcium score (GMCS) (548) 18 F-NaF heart uptake (top) compared to subject with low GMCS (291) 18 F-NaF heart uptake (bottom). The patient displays greater radiotracer uptake in the PET images (B and E) and PET/CT fusion images (C and F), indicated by colored pixel intensity. (Reproduced from: Borges-Rosa J, J Nucl Cardiol Off Publ Am Soc Nucl Cardiol. 2022 Aug;29 [4]:1846–54, with permission from Elsevier) [32]

Multiple analytical strategies have been employed in coronary arterial PET studies. Global assessment is one such method that focuses on whole heart segmentation, while other studies more narrowly focus on the coronary arteries, sometimes restricting their analysis to individual atherosclerotic plaques. Global assessment assumes little to no ^18^F-NaF uptake in the myocardium, enabling whole heart segmentations that represent arterial radiotracer uptake [30]. This approach has several advantages, including that it does not require administration of intravenous iodinated contrast material (which may be contraindicated in patients with renal insufficiency/failure or with prior allergic reaction), uses a large area of interest that mitigates the limitations of PET spatial resolution, and encompasses the small caliber arteries in addition to the main branches of the coronary arteries. A study by Sorci et al. used a global assessment approach on 136 subjects and demonstrated a statistically significant difference in ^18^F-NaF SUVmean of the heart between patient (*n* = 50) and healthy control (*n* = 89) groups (ages 23–75) [31]. SUVmean also showed a strong correlation with age and body mass index (BMI), both major risk factors for atherosclerosis. The same study also calculated calcium scores from CT images, with no significant difference being observed between the two subject groups.Borges-Rosa et al. assessed ^18^F-NaF uptake in 34 patients with elevated cardiovascular risk (mean age 63.5 ± 7.8 years) [32]. Global molecular calcium score (GMCS) was used to evaluate whole heart ^18^F-NaF activity and to compare subjects with less than 5 risk factors and those with more than 5 risk factors (Fig. 3). There was a significant difference in mean GMCS between the two groups, although no such difference was observed when target to background ratio (TBR) analysis, in this case a target SUVmax divided by a blood pool SUVmean typically taken from the superior or inferior vena cava, was applied. Associations of mean GMCS with BMI, waist circumference, and epicardial adipose tissue volume measured on CT were also found.

Methods of assessing focal uptake in the coronary arteries themselves pose greater challenges as the coronary arteries have a smaller diameter than the spatial resolution of existing PET/CT machines. Further complicating measurements is the constant movement of the coronary arteries caused by heart contractions and respiration. Cardiac gating techniques, which use only the data acquired during certain phases of the cardiac cycle, are employed in most studies to minimize the effect of cardiac contractions on the resulting images. There have also been studies assessing the viability of respiratory gating, but research has shown this area to be problematic [33, 34]. A study of 50 patients by Kwiecinski et al. used 3D regions of interest (ROI) around the coronary arteries to calculate the maximum TBR (TBRmax) of ^18^F-NaF maximum standardized uptake value (SUVmax) and coronary microcalcification activity (CMA), which is the integrated activity in SUV units exceeding the background blood pool SUVmean + 2 standard deviations [35]. The study then compared the TBRmax and CMA of vessels in patients with and without low attenuation plaque (LAP, with attenuation < 30 Hounsfield units) present. Both TBRmax and CMA were significantly higher in vessels with LAP present (as well as within patients), with CMA significantly outperforming TBRmax. A separate study by Kwiecinski et al. of 293 patients with advanced CAD followed subjects over 42 months and indicated the ability of CMA to predict major adverse cardiovascular events (MACE) (hazard ratio: 7.1; 95% CI: 2.2 to 25.1; *p* = 0.003), with no myocardial infarctions occurring in the subjects without increased ^18^F-NaF uptake in the coronary arteries [36]. Other groups have also demonstrated the prognostic utility of ^18^F-NaF PET. For example, in a study of 40 patients, Kitagawa et al. found M-TBRmax (a per patient maximum TBRmax of all separately evaluated lesions, which are well below the spatial resolution of PET) to be a predictor of MACE (hazard ratio, 5.4; 95% CI, 1.1–25.4; *P* = 0.034) [37]. These findings support a possible clinical use for ^18^F-NaF PET/CT but will require further prospective studies to validate and address concerns regarding the accuracy of targets below the spatial resolution of PET/CT machines.

### ^18^F-NaF PET for Thoracic Aortic Atherosclerosis

**Fig. 4:**
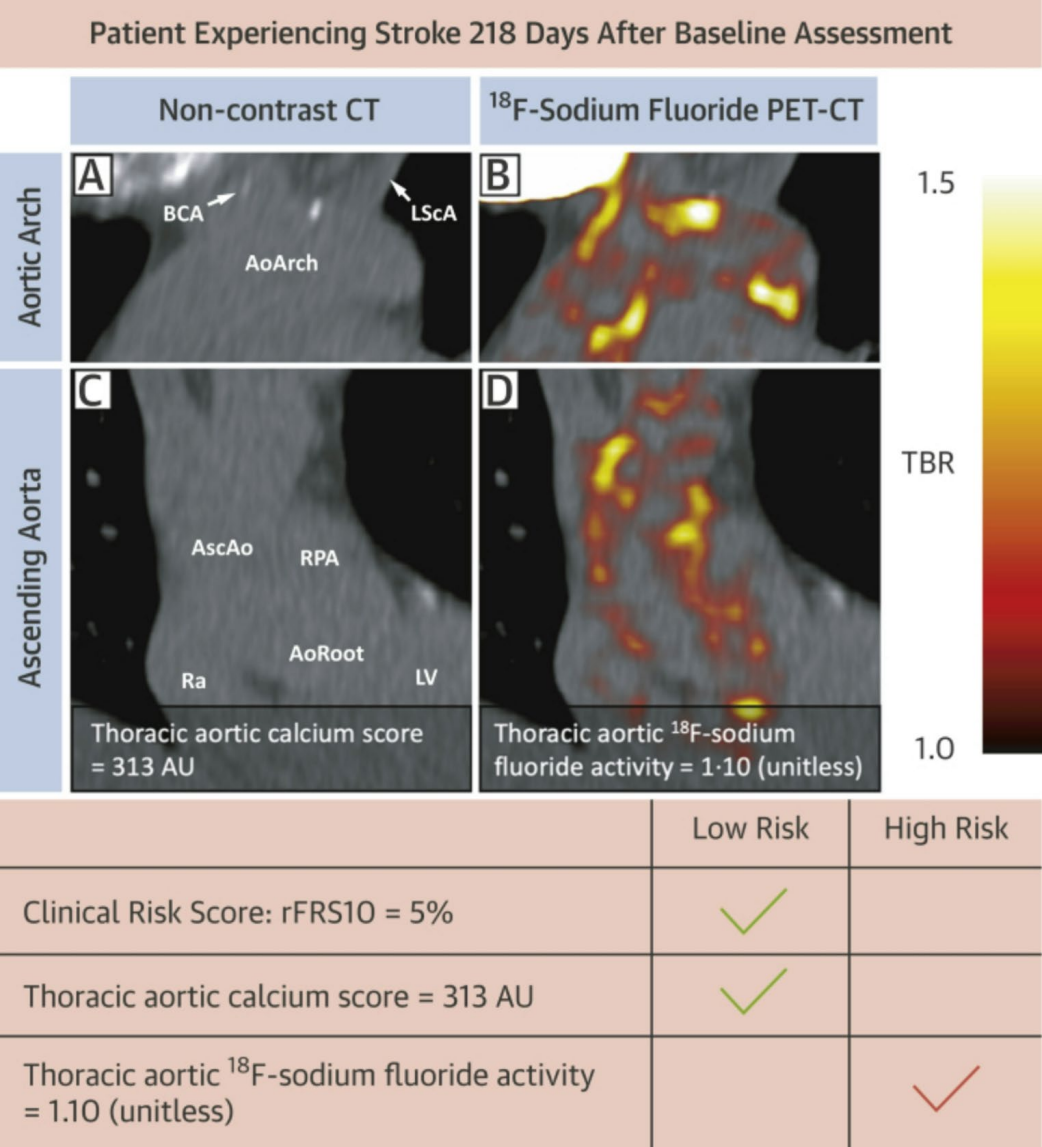
An example case demonstrating the ability of 18 F-NaF to supplement stroke risk prediction. In a 60-year-old patient with moderate aortic stenosis on aspirin and clopidogrel, both the clinical risk score and calcium score were low risk, but high 18 F-NaF uptake was observed. The patient went through bilateral posterior circulation infarcts 218 days after the initial scan, although no plaques were visible in the carotid arteries and there was no atrial fibrillation. AoArch = aortic arch; AoRoot = aortic root; AscAo = ascending aorta; AU = Agatston units; BCA = brachiocephalic artery; LScA = left subclavian artery; LV = left ventricle; Ra = right atrium; rFRS10 = Revised 10-year Framingham Stroke Risk Score; RPA = right pulmonary artery(Reproduced from: Fletcher AJ, et al. JACC Cardiovasc Imaging. 2022 Jul 1;15 [7]:1274–88, with permission from Elsevier) [42]

The thoracic aorta is also an intensely researched region of interest for ^18^F-NaF PET/CT studies. Atherosclerosis in this region represents a major risk factor for stroke and other arterial embolization. The thoracic aorta is also a much larger structure than the coronary arteries and is less susceptible to motion artifacts, making it a much more reliable structure to assess in PET images [38]. In a study of 124 subjects, 44 with chest pain syndromes and 80 healthy controls, Paydary et al. quantified thoracic aorta ^18^F-NaF uptake with the ACS and found it to be a significant predictor of an unfavorable CVD risk profile (odds ratio = 1.006, 95% CI = 1.000–1.013, *p* = 0.05) [39]. The study also demonstrated strong correlations between the ACS and the 10 year CVD risk using Framingham risk score (FRS), SUVmean, and age. Fletcher et al. proposed an aortic microcalcification activity (AMA) method, dividing thoracic aorta radiotracer activity (Bq/cm^3^) by left atrium radiotracer activity (Bq/cm^3^) [40]. Direct correlations between AMA and Framingham stroke risk score and FRS for hard coronary heart disease (defined in the Framingham Heart Study as myocardial infarction, coronary insufficiency, and coronary heart disease death [41]) were found in their analysis of 20 patients. A separate study by Fletcher et al. of 461 patients with a 12-month follow-up correlated AMA with thoracic aorta calcium volume progression measured from CT (*r* = 0.31) [42]. The findings also indicated that high AMA (> 1.1) was strongly correlated with risk of ischemic stroke (HR: 10.3 [95% CI: 3.1–34.8]; *p* = 0.00017) (Fig. 4), but not with the risk of myocardial infarction, and that baseline thoracic aortic calcium score was also associated with risk of ischemic stroke (log[thoracic aortic calcium score + 1]: HR: 1.27 [95% CI: 1.04–1.56]; p *=* 0.017). The use of ^18^F-NaF PET for assessment of the thoracic aorta demonstrates potential as a sensitive marker of microcalcific aortopathy and its long-term sequelae, including stroke.

### Applications for Therapeutics

There are several critical advantages of ^18^F-NaF PET/CT imaging for detection of atherosclerotic calcification over currently used structural imaging techniques. The first is the ability to visualize early-stage pathophysiologic changes prior to the onset of detectable morphologic findings on CT alone. The second is the potential to discern high-risk atherosclerotic plaques with active microcalcification from stable plaques. The early detection of clinically actionable atherosclerosis allows for early interventions through lipid-lowering therapeutics and lifestyle modifications, thereby slowing the progression of the disease and the likelihood of adverse sequelae. Such detection is also relevant to understanding the molecular effects of therapeutic agents aimed at reducing atheroburden.

**Fig. 5:**
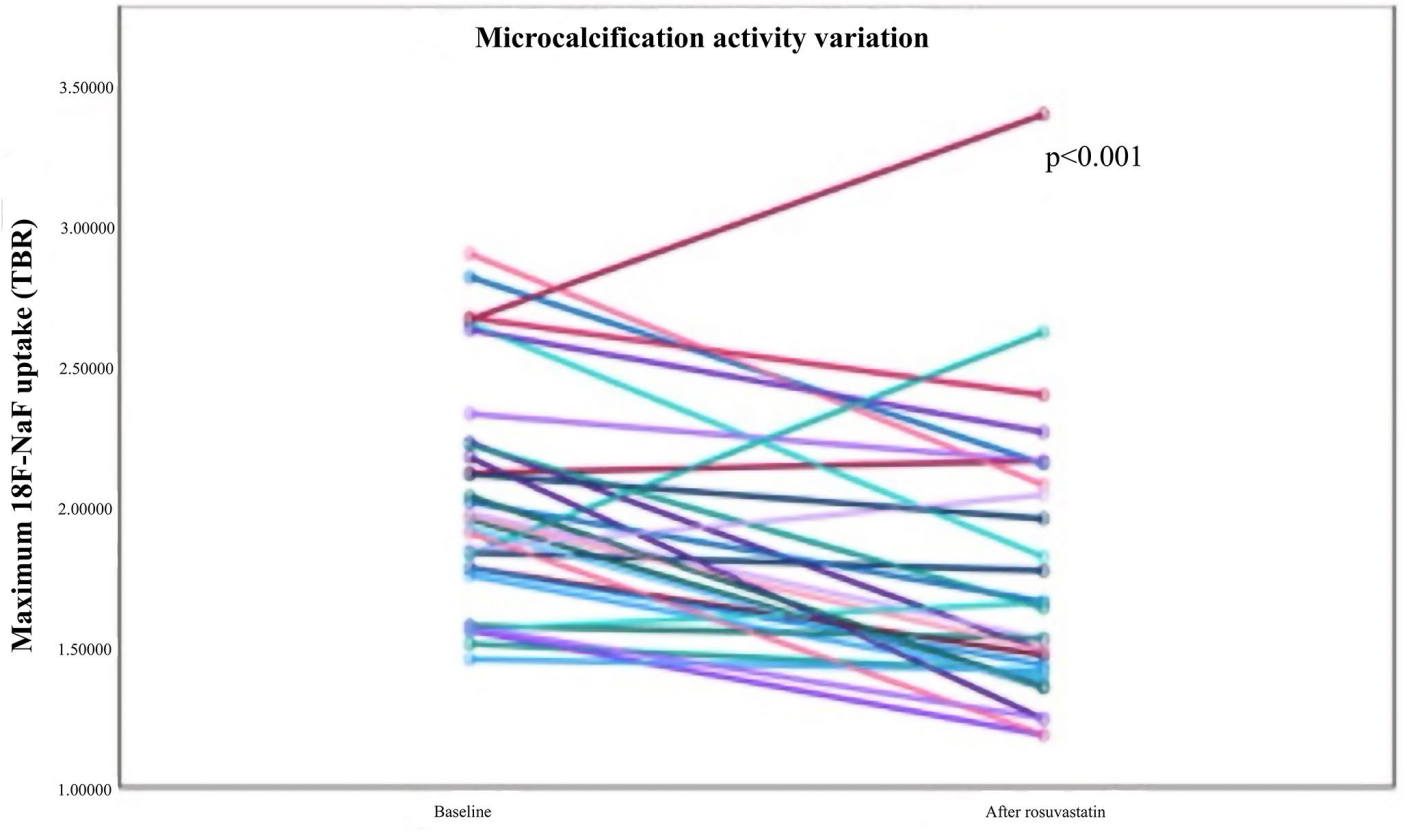
The figure shows maximum 18 F-NaF uptake before and after high intensity statin treatment, showing a large decrease in plaque activity in most subjects. (Reproduced from: Oliveira-Santos M, et al. Atherosclerosis [Internet]. 2024 Aug 1 [cited 2024 Aug 5];395, with permission from Elsevier) [44]

Statin therapy is a fundamental component of primary and secondary CVD prevention, principally recognized for its profound cholesterol-lowering effects. Growing evidence has further indicated a beneficial effect of statin therapy through cholesterol-independent or pleiotropic mechanisms, including atheroma stabilization [43]. ^18^F-NaF PET imaging reveals insight into the relationship between statin use and microcalcification, thus serving as a potential biomarker of therapeutic efficacy. One study by Oliveira-Santos et al. demonstrated a 19% reduction in ^18^F-NaF uptake of atherosclerotic plaques on PET images after 6 months of rosuvastatin therapy in 38 individuals with subclinical atherosclerosis [44] (Fig. 5). A separate study by Puri et al. found that statin therapy correlated with increased calcification as well as plaque size reduction as assessed by IVUS [45]. Both studies reveal insight into the plaque stabilizing effect of statin therapy, indicating that lipid-lowering treatment can slow the progression of atherosclerosis and even stabilize already formed plaques, making it one of the most potent therapeutic options available.

### Different Strategies of Image Analysis

Different methods of image analysis present unique advantages and disadvantages. Global assessment of atherosclerosis using ^18^F-NaF PET is well-suited for the role of risk stratification and statin therapy assessment. ^18^F-NaF uptake (based on SUVmean and ACS) in the studies of Sorci et al. and Paydary et al. correlated with risk factors of atherosclerosis, demonstrating significant differences in SUVmean between patients and controls that were not observed with CT-based calcium score [31, 39]. Similar results were obtained by Borges-Rosa et al. in an older high-risk subject group without symptomatic CAD, indicating the utility of global assessment to detect early-stage atherosclerosis [32].

The studies of Kwiecinski et al. implement a different approach with focal ROIs localized to the coronary arteries directly [35, 36]. Their analyses of older patients with advanced CAD indicate promise in the role of ^18^F-NaF PET for MACE prediction, possibly aiding clinicians with proper management of CAD with antiplatelet therapy. However, the use of focal ROIs may have reduced the accuracy of the measurements on account of the partial volume effect and cardiac motion. Another limitation of these studies is their reliance on TBR or SUVmax as quantitative metrics of ^18^F-NaF uptake, both of which have proven to be unreliable metrics [26, 46]. SUVmax focuses on the most active pixel in the ROI, which is easily influenced by spillover from surrounding structures and may not reflect fully the characteristics of a given lesion or structure of interest [47], which may lead to insignificant results as shown by Sorci et al. [30]. TBR has also been proven problematic, yielding inconsistent values at different acquisition times of the PET image relative to the timing of radiotracer administration. Obtaining the blood pool activity value, the denominator of TBR, relies on measurement from venous compartments such as the superior vena cava, inferior vena cava, or right atrium, with the assumption that estimates of blood activity are comparable among vessels. However, studies have demonstrated significant variability in blood activity among different vascular beds, such that use of TBR may further exacerbate errors in quantification [48]. This underscores the need for a more standardized and rigorously validated methodology, as most studies compare very different metrics, thereby hampering efforts to gauge reliability and compare study results.

The ROIs themselves, however, have proved to be highly reproducible. A study by Piri et al. demonstrated strong intra- and inter-operator agreement on measures of ^18^F-NaF uptake from segmentations of the global cardiac ROI (Table 1) [50]. The consistent values obtained from ^18^F-NaF PET/CT quantification, particularly via global SUVmean, support the clinical application of this radiotracer in the assessment of atheroburden if such objective methodology can be implemented. Moreover, artificial intelligence (AI)-based quantification of ^18^F-NaF uptake achieved values consistent with the manual approach, showcasing the utility of this advanced technique for further improving reproducibility of ^18^F-NaF PET/CT quantification.

**Table 1:**
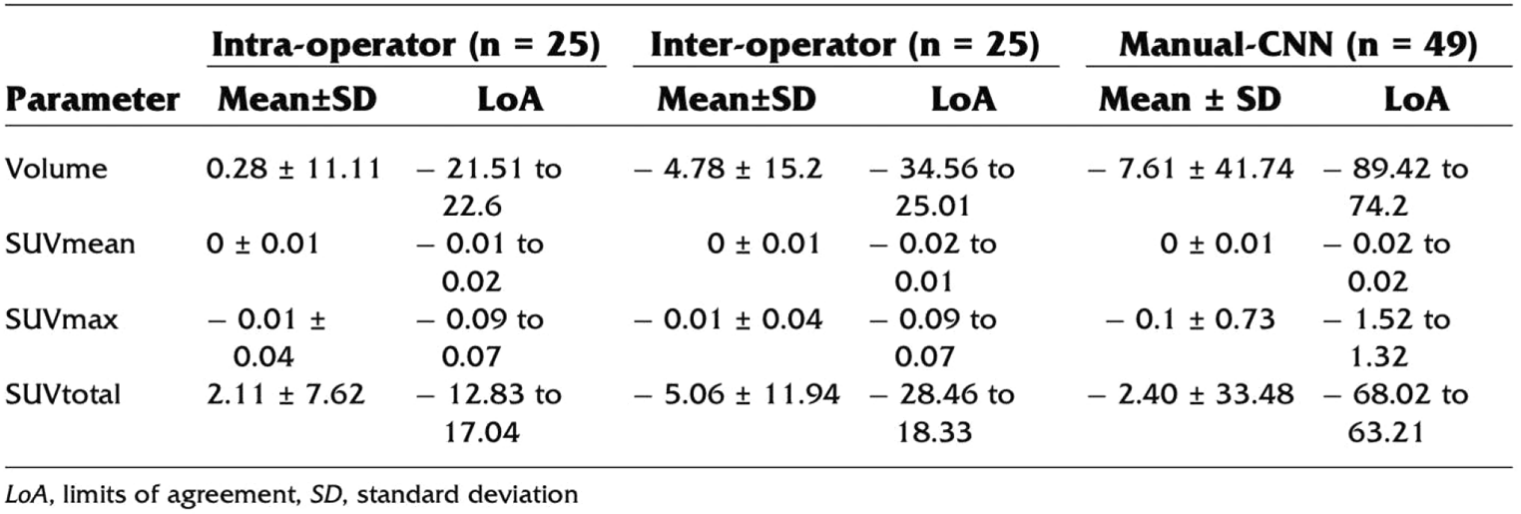
Shows the mean difference between different segmentations of the heart, assessing intra/inter-operator variability, and the mean difference between a manual segmentation and a convolutional neural network. The average manual segmentation values were for volume 617.65 ± 154.99, SUVmean 0.69 ± 0.15, SUVmax 2.68 ± 0.86, SUVtotal 425.51 ± 138.93. Intra-operator variability in vol, SUVmean, SUVmax and SUVtotal differed by 0–0.5%. Inter-operator variability in vol, SUVmean, SUVmax and SUVtotal differed by 0–1%. The manual and convolutional neural network (CNN) mean values for vol, SUVmax, and SUVtotal differed by 0–4%. (Reproduced from: Piri R, J nucl cardiol off publ am Soc nucl cardiol. 2022 Oct;29 [5]:2531–9; with permission from Elsevier) [54]

### The Potential of Artificial Intelligence in PET/CT

Artificial intelligence (AI) is a rapidly evolving domain with relevant applications for cardiovascular PET imaging. Automated segmentation of structures of interest can provide a time-efficient and reproducible method of quantifying radiotracer uptake, addressing aforementioned concerns on the reliability of different manual approaches. Several studies from Piri et al. have validated the use of AI-based segmentation to quantify ^18^F-NaF uptake in various vascular beds, obtaining values comparable to manual segmentation in the heart, aortic wall, and common carotid arteries [49–51]. The ability of AI-based segmentation to quantify ^18^F-NaF uptake on PET images in vessels of different size and anatomic variability is a promising development that requires further validation on external datasets.

The combination of AI with the extraction of high-throughput imaging features from PET/CT data via radiomics presents a novel development for risk assessment and patient management [52]. Radiomics provides quantitative features extracted from imaging data for particular structures of interest that may or may not be discernible to the human eye (e.g., shape, intensity, texture), which may potentially correlate with clinically relevant parameters of interest. For example, in the field of oncology, PET/CT radiomics has demonstrated significant utility as a method of improving tumor detection, tumor staging, and prediction of patient survival [53, 54]. Coronary CT radiomics can provide important detail on plaque phenotype and improve the estimation of future adverse cardiovascular events [55]. Implementation of radiomics-based AI may thus expand the application of ^18^F-NaF PET/CT for patients with CVD, providing diagnostic and prognostic detail on atherosclerosis progression that cannot be appreciated by conventional imaging methods alone.

## Conclusions

Atherosclerosis remains a leading cause of mortality worldwide. While the odds of surviving major adverse cardiovascular events have significantly improved with vascular interventions, the prevalence remains high. Current preventive measures rely on risk assessment using patient history (smoker status, average exercise, etc.) and laboratory tests, which although useful do not provide specific information about a patient’s risk for disease progression or optimal management. Current research has indicated that ^18^F-NaF PET/CT may be ideally suited for the role of risk assessment and therapy monitoring, allowing physicians to properly tailor management plans for individual patients. Yet, ^18^F-NaF PET/CT is missing standardized quantification techniques for atheroburden, and as such, further prospective studies are necessary to evaluate its clinical significance.

## Key References

- Borges-Rosa J, Oliveira-Santos M, Silva R, da Silva NP, Abrunhosa A, Castelo-Branco M, et al. Cardiac microcalcification burden: Global assessment in high cardiovascular risk subjects with Na[18 F]F PET-CT. J Nucl Cardiol Off Publ Am Soc Nucl Cardiol. 2022 Aug;29 [4]:1846–54.
-
  - The work by Borges-Rosa et al. continued with global molecular calcium score research, demonstrating advantages of^18^F-NaF PET/CT over current methods for assessing coronary arterial atherosclerosis.
- Fletcher AJ, Tew YY, Tzolos E, Joshi SS, Kaczynski J, Nash J, et al. Thoracic Aortic 18 F-Sodium Fluoride Activity and Ischemic Stroke in Patients With Established Cardiovascular Disease. JACC Cardiovasc Imaging. 2022 Jul 1;15 [7]:1274–88.
-
  - The study by Fletcher et al. strongly indicated the usefulness of ^18^F-NaF PET imaging for prognostic purposes, thereby paving the way for future prognostic studies.
- Oliveira-Santos M, Borges-Rosa J, Silva R, Paixão L, Santo CE, Abrunhosa A, et al. Rosuvastatin effect on atherosclerotic plaque metabolism: A subclinical atherosclerosis imaging study with 18 F–NaF PET-CT. Atherosclerosis [Internet]. 2024 Aug 1 [cited 2024 Aug 5];395. Available from: https://www.atherosclerosis-journal.com/article/S0021-9150(24)00041-8/fulltext.
-
  - The work by Oliveira-Santos et al. reported the results of statin therapy using ^18^F-NaF PET imaging, providing insight into the stabilizing effects of current atherosclerosis therapies and a potential method to monitor therapeutic effect.

## Data Availability

No datasets were generated or analysed during the current study.
